# Dental sealant knowledge, opinion, values and practice of Spanish dentists

**DOI:** 10.1186/1472-6831-13-12

**Published:** 2013-02-08

**Authors:** Laura San Martin, Antonio Castaño, Manuel Bravo, Mary Tavares, Richard Niederman, Eyitope O Ogunbodede

**Affiliations:** 1 School of Dentistry, University of Seville, Avicena s/n, Seville, Spain; 2 Forsyth Institute, Cambridge, MA, USA; 3 Harvard School of Dental Medicine Boston, Boston, MA, USA; 4 School of Dentistry, University of Seville, Seville, Spain; 5 School of Dentistry, University of Granada, Granada, Spain; 6 Forsyth Institute, Cambridge, MA, USA; 7 Harvard School of Dental Medicine, Boston, MA, USA; 8 Forsyth Institute, Cambridge, MA, USA; 9 Faculty of Dentistry, Obafemi Awolowo University, Ile-Ife, Nigeria

**Keywords:** Fissure sealants, Dental, Prevention, Children, Oral health

## Abstract

**Background:**

Multiple guidelines and systematic reviews recommend sealant use to reduce caries risk. Yet, multiple reports also indicate that sealants are significantly underutilized. This study examined the knowledge, opinions, values, and practice (KOVP) of dentists concerning sealant use in the southwest region of Andalusia, Spain. This is a prelude to the generation of a regional plan for improving children’s oral health in Andalusia.

**Methods:**

The survey’s target population was dentists working in western Andalusia, equally distributed in the provinces of Seville, Cadiz, and Huelva (N=2,047). A convenience sample of meeting participants and meeting participant email lists (N=400) were solicited from the annual course on Community and Pediatric Dentistry. This course is required for all public health sector dentists, and is open to all private sector dentists. Information on the dentist’s KOVP of sealants was collected using four-part questionnaire with 31, 5-point Likert-scaled questions.

**Results:**

The survey population demographics included 190 men (48%) and 206 women (52%) with an average clinical experience of 10.6 (± 8.4) years and 9.3 (± 7.5) years, respectively. A significant sex difference was observed in the distribution of place of work (urban/suburb) (p=0.001), but no sex differences between working sector (public/private). The mean ± SD values for each of the four KOVP sections for pit and fissure sealants were: knowledge = 3.57 ± 0.47; opinion = 2.48 ± 0.47; value = 2.74 ± 0.52; and practice = 3.48 ± 0.50. No sex differences were found in KOVP (all p >0.4). Independent of sex: knowledge statistically differed by years of experience and place of work; opinion statistically differed by years of experience and sector; and practice statistically differed by years of experience and sector. Less experienced dentists tended to have slightly higher scores (~0.25 on a Likert 1–5 scale). Statistically significant correlations were found between knowledge and practice (r=0.44, p=0.00) and between opinion and value (r=0.35, p=0.00).

**Conclusions:**

The results suggest that, similar to other countries, Andalusian dentists know that sealants are effective, have neutral to positive attitudes toward sealants; though, based on epidemiological studies, underuse sealants. Therefore, methods other than classical behavior change (eg: financial or legal mechanisms) will be required to change practice patterns aimed at improving children's oral health.

## Background

Dental caries is among the most common of preventable childhood infections [1], and methods are currently available to cost effectively reduce caries [2]. The most effective method to reduce occlusal caries are pit and fissure sealants, and over the last four years more than 11 guidelines and systematic reviews have recommended pit and fissure sealant use for at-risk populations [3-13]. However, studies from U.S. [14-16], Greece [17], Sweden [18], and Scotland [19,20] all indicate that sealants are underutilized.

In Spain, recent surveys indicate a 56% caries prevalence among 15–16 year olds, while only 17% have sealants [21,22]. Other Spanish studies demonstrate that occlusal sealants can reduce both occlusal and smooth surface decay by 87% and 68%, respectively, over a two year period [23]. Over a nine year period sealants can reduce occlusal decay by 65% [24].

Thus there are effective methods for caries prevention, but they are underutilized. The theoretical frame for behavior change is an assessment of knowledge and attitudes affecting practice. However, neither theories of behavior change nor knowledge nor attitudes predict clinical practice [25]. Instead, both indicate that values are better predictors [20,26]. Therefore, we examined knowledge combined with opinions and values, as a first step toward initiating comprehensive caries prevention program in Spain. More particularly we assessed dentists in the western province of Andalusia regarding to the use of pit and fissure sealants.

## Methods

### Literature search

A comprehensive search for meta-analysis and systematic reviews on pit and fissure sealants was conducted by the authors using the Pubmed Database (http://www.ncbi.nlm.nih.gov) (Tables 1 and 2).

**Table 1:** To identify the current best evidence on pit and fissure sealants we queried MEDLINE using the following search strategy

**Step**	**Search**	**Found**
1	(dental sealants) OR (tooth sealants) OR (fissure sealants) OR (pit and fissure sealants)	3058
2	(meta-analysis) OR (systematic review)	1,732,591
3	1 AND 2	348
4	2008[PDat]:2012[PDat]	3,756,585
5	3 AND 4	68
	Relevant = 11	

Search #1 was limited to systematic reviews of human randomized controlled trials. The titles and abstracts were examined to identify systematic reviews with meta-analysis relevant to caries prevention.

**Table 2:** To identify the current best evidence on knowledge, attitudes and practice regarding pit and fissure sealants we queried MEDLINE using the following search strategy

**Step**	**Search**	**Found**
1	(dental sealants) OR (tooth sealants) OR (fissure sealants) OR (pit and fissure sealants)	3058
2	(Health care quality, access, evaluation) OR (patient care management) OR (behavior and behavior mechanisms)	2,228,959
3	1 AND 2	318
4	2008[PDat]:2012[PDat]	3,756,585
5	3 AND 4	78
	Relevant = 7	

Search #2 was limited to surveys of on KAP regarding sealants. The titles and abstracts were examined to identify clinical trials relevant to pit and fissure sealants.

### Survey generation

Information from the studies identified in the MEDLINE searches were used to generate the 31 survey questions in four groups: knowledge, opinion, values and practice. Each question was reviewed for pertinence and clarity by 8 full and part-time faculty who teach prevention (N=5), pediatric dentistry (N=2), or are program directors for community dentistry (N=1) in Andalusia. A Likert 1–5 scale survey (1 = strongly disagree; 5 = strongly agree), and narrative commentary were used to evaluate the survey. The questions were edited as suggested by the initial survey panel. The survey was then sent to 20 dentists twice, one week apart, to determine test/retest validity.

### Survey method

The survey’s target population was dentists working in 3 of the 4 western provinces of Andalusia: Seville, Cadiz, and Huelva (N=2,047). Raosoft was used to generate a power calculation (http://www.raosoft.com/samplesize.html). For a 5% margin of error, a 95% confidence level, a population of 2,047, and a response distribution of 50%, the minimum recommended survey size is 324.

We solicited a convenience sample of participants from the annual Community and Pediatric Dentistry course. This is a required course for all public health sector dentists, and is open to all private sector dentists. It is attended by dentists with an interest in pediatric dentistry. Direct solicitation of meeting participants and solicitation from participant email lists were used to recruit a convenience sample of 400 dentists, equally distributed between the three provinces. Meeting participants were surveyed at the meeting, in person, and accounted for approximately 46% of the responses. The meeting participants provided email addresses during registration. From this email list we selected individuals who did not respond in person. From this email list we identified individuals from three geographic regions. We then emailed the identified number of individuals in each region asking them if they would complete the survey. We iteratively continued this until we filled the quota determined by the power calculation. The total number of professionals attending the meeting who responded to our survey was 184. The remaining 216 participants were recruited by email.

### Demographics

Survey respondents were identified by 4 metrics: sex, years in practice (≤ 3, 4–15, ≥ 16), practice location (urban, suburban), and type of practice (public, private, both).

### Statistical analysis

For each of the 31 survey items and for each of the four assessment domains (knowledge, opinions, values, and practice) a frequency distribution of the Likert scale was determined as well as the mean. These were evaluated individually using the Wilcoxon matched-pairs signed-ranks test, the Friedman nonparametric repeated measures ANOVA, or Spearman’s correlation.

## Results

### Survey

Test-retest validity was determined with 20 dentists retested at an interval of one week (Cronbach´s alpha =0.872). The frequency distribution for the 31 questions in the four sections - knowledge, values, opinion, and practice - is presented in Table 3. The mean ± sd scores for the individual questions ranged from 1.53 ± 0.82 to 4.29 + 0.90 with an overall of 3.15±0.31. All the dentists who attended the course filled out our survey. Our response rate from the emails was 76%.

**Table 3:** Distribution and mean ± standard deviation of scores for the questions

**QUESTIONS**		**Percentages**					*Mean ± SD
		**1**	**2**	**3**	**4**	**5**	
		**Strongly disagree**	**Disagree**	**Neutral**	**Agree**	**Strongly agree**	
**Knowledge**							
Q1	I think that the effectiveness of fissure sealants is supported by strong scientific evidence.	5.5	8.5	21.8	27.2	37.0	3.82±1.18
Q2	There is scientific evidence for the restorative use of dental sealants.	7.0	15.5	35.5	22.2	19.8	3.32±1.16
Q3	I am familiar with the technique of placing dental sealants.	6.8	3.8	14.8	33.5	41.2	3.99±1.15
Q4	I believe that fissure sealants should be reviewed after placement.	1.8	2.0	14.0	30.0	52.2	4.29±0.90
Q5	I understand the instructions for placing sealants.	1.8	3.0	26.0	27.2	42.0	4.05±0.98
Q6	I think that sealants should only be used on newly erupted teeth	21.8	14.2	21.5	20.0	22.5	3.07±1.45
Q7	I think that sealants wear out easily.	6.0	17.5	32.8	26.2	17.5	3.32±1.13
Q8	I believe that you must perform a caries risk assessment to prevent overtreatment.	4.8	3.0	26.2	34.2	31.8	3.85±1.05
Q9	Pit and fissure sealants have adverse effects.	28.5	21.5	25.0	14.8	10.2	2.57±1.31
Q10	I believe the technique of applying a sealant is the most important aspect to the success of the treatment.	9.5	10.8	28.0	28.8	23.0	3.45±1.22
Q11	I agree that resin sealants are more effective than glass ionomer sealant.	7.6	13.0	33.6	27.0	18.8	3.36±1.15
Q12	The most important factor for adhesion to occur in sealant placement is proper acid etching.	6.8	5.5	26.2	34.8	26.7	3.69±1.13
**Value**							
Q13	I think this technique takes time to do correctly.	39.0	35.2	19.5	5.8	0.5	1.94±0.92
Q14	The materials that are used for the placement of sealants are very expensive.	23.1	38.7	25.1	10.8	2.3	2.30±1.01
Q15	I do not use sealants very often as a preventive method because its effect is short lived.	25.0	28.2	18.0	19.5	9.2	2.60±1.30
Q16	Fissure sealants are used less than they should be.	9.5	6.8	27.1	31.4	25.1	3.56±1.21
Q17	The dental staff at my clinic communicate importance of using sealants to the patients.	8.9	13.5	36.1	19.6	21.9	3.32±1.21
**Opinion**							
Q18	It is difficult to explain to patients what dental sealants are.	25.5	21.2	21.8	25.8	5.8	2.65±1.27
Q19	It is difficult to justify the cost of sealants to parents	15.8	18.3	31.3	22.6	12.0	2.97±1.24
Q20	I think my patients understand the benefits of using sealants.	28.0	23.0	31.1	9.3	8.6	2.47±1.23
Q21	It is necessary to promote the use of sealants amongst dentists and dental educators.	4.0	11.8	19.3	30.6	34.3	3.79±1.15
Q22	I apply sealants because the oral public health community instructs me to.	55.9	23.5	13.6	4.7	2.3	1.74±1.02
Q23	I use dental sealant in the oral public sector because it is easy to apply and patients find it comfortable.	66.5	15.8	16.3	1.5	0.0	1.53±0.82
Q24	Since working in the oral health public community, I have greater belief in the effectiveness of sealants.	42.9	18.4	25.9	8.0	4.7	2.13±1.19
**Practice**							
Q25	I sometimes avoid dental sealants for the possibility of sealing over caries.	14.2	15.0	23.0	22.8	25.0	3.29±1.37
Q26	I think sealants, besides being a preventive method, can also have a restorative effect and can be used on incipient caries.	35.6	25.1	17.8	13.3	8.3	2.34±1.30
Q27	This sealing technique, when used alongside fluoride application, may reduce the rate of decay more significantly.	5.5	8.0	30.5	24.5	31.5	3.68±1.16
Q28	In the case of partial or total loss of sealant, I would recommend reapplication.	6.8	13.1	21.2	25.9	33.0	3.65±1.25
Q29	The most important factor for adhesion to occur in sealant placement is proper insolation.	2.5	5.0	17.6	37.8	37.0	4.02±0.99
Q30	The most important factor for adhesion to occur in sealant placement is proper acid etching.	4.0	5.3	27.9	38.9	23.9	3.73±1.01
Q31	The benefits of using sealants should be considered with regard to the patient’s risk of caries and clinicians should follow specific guidelines	2.8	8.1	38.5	25.9	24.7	3.62±1.03

### Demographics

A target population of 400 dentists was selected from a larger population of 2,047 dentists working in Western Andalusia. The dentists were equally recruited from the three provinces of Seville (34%), Cadiz (33%) and Huelva (33%). For the 400 dentists who provided opinion data 396 provided demographic data (Table 4). These were divided between male and female as 190 (47.5%) and 210 (52.5%), respectively. They had 10.59±8.39 and 9.26±7.52 years of experience, respectively (P =0.095). The mean (±SD) years of experience for the total respondents was 9.20±7.97 (range 0 to 37) years. Two hundred and sixty-one of respondents (65%) worked only in private clinics, 46 (12 %) in public sector clinics and 89 (22%) in both private and public sector clinics. There was no difference between both sexes (P =0.114). One hundred and thirty-two respondents (33%) worked in urban settings, 177 (44%) in suburban settings, and 87 (22%) in both. There was a significant sex difference in the distribution (P =0.001). While 27 (31%) of 190 males worked in both urban and suburban clinics, the corresponding figure for females was 60 of 206 (69%) (Table 4).

**Table 4:** Demographics

			**Sex**						
			**Male**			**Female**			
		**N**	**Mean ± SD**	**n**	**(%)**	**Mean ± SD**	**n**	**(%)**	**P**
		396		190	48.0		206	52.0	
**Years of experience**			10.59±8.39			9.26±7.52			0.095
Sector	Public	46		28	(60,9)		18	(39,1)	0.114
	Private	261		117	(44,8)		144	(55,2)	
	Both	89		45	(50,6)		44	(49,4)	
Place of work	Urban	132		74	(56,1)		58	(43,9)	0.001
	Suburb	177		89	(50,3)		88	(49,7)	
	Both	87		27	(31,0)		60	(69,0)	

KOVP. Knowledge, value, opinion and practice around pit and fissure sealants were examined, first as groups and then by demographics. As a group the average values were: knowledge = 3.57 ± 0.47; opinion = 2.48 ± 0.47; practice = 3.48 ± 0.50; and value = 2.74 ± 0.52. The groups were then segregated by four metrics: sex, years of experience, practice sector and place of work (16 total assessments) (Table 5). The scores within the 16 metrics ranged from 2.37 to 3.67 (1=strongly disagree; 5=strongly agree), indicating a neutral to positive impression of pit and fissure sealants.

**Table 5:** Summary table for knowledge, values, opinion, practice

		**Knowledge (Q 1–12)**	**Value (Q 13–17)**	**Opinion (Q 18–24)**	**Practice (Q 25–31)**	**Total (Q 1–31)**
**Mean±SD**						
**1**	**Sex**					
	Male (n=187)	3.55±0.42	2.76±0.51	2.50±0.42	3.47±0.50	3.14±0.30
	Female (n=204)	3.58±0.52	2.73±0.53	2.45±0.51	3.48±0.50	3.15±0.31
	Total (n=391)	3.57±0.47	2.74±0.52	2.48±0.47	3.48±0.50	3.15±0.31
		**P=0.44**	**P=0.55**	**P=0.41**	**P=0.82**	**P=0.76**
**2**	**Years of Experience**					
	≤3 (n=38)	3.64±0.33	2.79±0.52	2.61±0.53	3.47±0.47	3.17±0.28
	4-15 (n=89)	3.60±0.43	2.73±0.51	2.49±0.41	3.57±0.46	3.19±0.28
	≥16 (n=57)	3.42±0.63	2.72±0.55	2.37±0.49	3.30±0.56	3.06±0.35
	Total (n=184)	3.57±0.47	2.74±0.52	2.48±0.47	3.48±0.50	3.15±0.31
		P=0.00*	**P=0.71**	P=0.03*	P=0.00*	P=0.03*
**3**	**Sector**					
	Public	3.67±0.33	2.78±0.35	2.39±0.38	3.48±0.44	3.18±0.25
	Private	3.56±0.50	2.72±0.54	2.35±0.51	3.49±0.51	3.06±0.38
	Both	3.52±0.46	2.81±0.53	2.65±0.42	3.43±0.51	3.21±0.24
	Total	3.57±0.47	2.74±0.52	2.48±0.47	3.48±0.50	3.15±0.31
		**P=0.23**	**P=0.39**	P=0.00*	**P=0.62**	P=0.01*
**4**	**Place of Work**					
	Urban	3.43±0.56	2.73±0.50	2.46±0.53	3.40±0.54	3.05±0.34
	Sub-urban	3.65±0.42	2.79±0.55	2.47±0.42	3.50±0.47	3.22±0.28
	Both	3.58± 0.36	2.69±0.49	2.52±0.45	3.52±0.49	3.18±0.25
	Total	3.57±0.47	2.74±0.52	2.48±0.47	3.48±0.50	3.15±0.31
		P=0.00*	**P=0.33**	**P=0.77**	**P=0.13**	P=0.00*

*Significant difference.

As indicated in Table 5, of the 16 assessments, 5 demonstrated statistically significant differences: Knowledge differed by years of experience (p=0.00), and place of work (p = 0.00); Opinion differed by years of experience (p=0.03) and sector (p=0.00). Similarly, practice about sealants differed by years of experience (p=0.00). Conversely, values around sealant placement did not statistically differ by sex, years of experience, practice sector or place of work.

The correlations between knowledge, opinion, values and practice is presented in Table 6. Statistically significant correlations were found between knowledge and practice (r=0.44, p=0.00) and between value and opinion (r=0.35, p=0.00).

**Table 6:** Relationship between mean knowledge, values, opinions and practice

	**Knowledge**	**Value**	**Opinion**	**Practice**
	**Spearman’s correlation**			
**Knowledge**	1.000	0.054	0.056	0.439^**^
	.	P=0.287	P=0.441	P=0.000
	(N= 385)	(N= 385)	(N=192)	(N=384)
				
**Value**	0.054	1.000	0.351^**^	0.002
	P=0.287	.	P=0.000	P=0.966
	N=385	(N=390)	N=191	N=383
**Opinion**	0.056	0.351^**^	1.000	−0.003
	P=0.441	P=0.000	.	P=0.965
	N=192	N=191	N=194	N=189
**Practice**	0.439^**^	0.002	−0.003	1.000
	P=0.000	P=0.966	P=0.965	.
	N=384	N=383	N=189	N=390

** Correlation is significant at the 0.01 level (2-tailed).

## Discussion

These primary findings indicate that dentists in western Andalusia have neutral to favorable knowledge, opinion, values, and practice attitudes about sealants (Table 3). The more detailed statistical findings for KOVP in relationship to demographics (Tables 5 and 6) need to be interpreted with caution. First, some are, and some are not significant. Yet, for those that are significant, the absolute differences between the highest and lowest values in each category for KOVP are modest. Second, these are secondary outcomes of association, and should be viewed as hypothesis generating. The study was powered for the primary outcome variables, not the secondary outcome variables. Therefore, the analyses in Tables 5 and 6 are vulnerable to type 1 or type 2 errors. In sum, the findings reported here: (1) adds Spain to a list of other countries in what appears to be a growing global phenomenon of under-utilization of the preventive sealants [14-19,27]; and (2) expands the list of behavioral metrics around dental sealant knowledge and attitudes to include opinions and values that do not appear to adequately relate practice behavior. The survey did not, however: (1) determine the relationship of KOVP to actual sealant use; or (2) determine the relationship of traditional KAP with KOVP.

A troubling overall finding is the recognition of a clinical problem (caries), the availability of a cost-effective preventive solution (sealants), a neutral to positive KOVP, and the reluctance of the professional community to implement sealants in practice. This suggests that behavioral phenomena other than or in addition to KOVP may be driving clinical practice.

One hypothesis is that the theoretical KOVP imparted during dental school training differs from clinical training. For example, over the last 100 years dentists have successfully treated caries with surgery and repair using silver amalgam, plastic, gold or stainless steel. Dental schools therefore focus on providing training to instill basic knowledge of anatomy, anesthesia, material science, and clinical skill to facilitate surgical expertise in repairing the cavitated lesion. In parallel, compensation systems have evolved to reward clinicians for providing surgical care. This system therefore rewards treatment over prevention.

However, for more than 50 years, the oral health community has recognized that caries is a preventable infection, and developed methods to reduce the probability of initiation and progression of the infection. The most effective preventive method for occlusal caries is the application of pit and fissure sealants to the occlusal surfaces [3-13], which can reduce dental decay by up to 80%. Yet, globally, the epidemic of caries continues unabated [1,2].

From a global oral health perspective, effecting systematic change in clinical care delivery to support prevention is challenging [14-19,27]. This is also true historically across the health spectrum, ranging from the use of vitamin C to prevent scurvy (250 years before routine implementation) [28], to hand washing (100 years before routine implementation) [29], to a host of interventions and diagnostics that over treat, undertreat or mistreat patients (more than 15 years for routine implementation) [30].

From a behavioral viewpoint, multiple models have been suggested to effect behavior change. [31,32]. Pioneering work by Bonetti and colleagues [20,27,33] examined six theoretical models for behavior change around sealant use. They include: action planning, common sense self-regulation model, operant learning theory, the precaution adoption process, social cognitive theory, and the theory of planned behavior. Their examination included knowledge as an additional predictor. Using multiple regression analysis, they found that these models for behavior accounted for only 38% in the variation in care. This again suggests that the current models for effecting clinical practice behavior change do not go far enough in identifying significant levers for change. Their work does suggest, however, that economic and legal mechanisms may be more effective in altering clinical practice.

In Spain, the National Health System provides care for all children, independent of parental income. State or regional programs can supplement this care. In Andalusia, two frameworks for oral care are available: public and private. Private dentists receive 36 euros for providing care. In contrast, dentists working in the public sector are on salary and remuneration is independent of the type of care they provide. This compensation system for the private sector may be a significant factor impacting the selection of care delivered. Similarly, the cost of materials (fluoride varnish, glass ionomer, composite, and amalgam) can impact the selection of care in the public sector since funds for purchasing are limited. Thus, economics, not KOVP, may be the practice drivers in Spain. Interestingly, Clarkson et al. examined the economic/education/values hypothesis. They found that only economics altered practice behavior by dentists [20]. The current findings that KOVP are not predictive of practice, combined with the findings of Clarkson et al., suggest that alternate approaches to practice change and improving children’s oral health are needed.

Work at the Harvard Business School suggests that focusing on values may be the key driver for health improvement [34-36]. This group postulates and provides examples of systems that focus on rewarding value (defined as outcomes/costs for the care cycle). These systems increase access to care, improve health, and reduce the costs of care. The systems the Harvard team examined who use a values approach have: increased access to care, improved health, and reduced the costs of care. The Commonwealth Fund, in an extensively documented report, supports a value-based approach to care improvement [37]. Further support for this concept, as it applies to oral health improvement in the U.S. comes from the Pew Trust report [38]. The Pew Trust found that sealants are underused, resulting from two significant barriers to care: legal and economic.

In sum, the improvement of oral health, through the increased use of sealants, does not appear to be related to improving knowledge, attitudes, opinion or values of the dental community. Rather, economic and legal variables appear to provide more leverage. The oral health community must now consider if, when, and how we might begin to test these new hypothesis.

## Conclusions

The results reported here suggest that, similar to other countries, Andalusian dentists know that sealants are effective, have neutral to positive attitudes toward sealants; though, based on epidemiological studies, underuse sealants. Therefore, methods other than classical behavior change (eg: financial or legal mechanisms) will be required to change practice patterns aimed at improving children's oral health.

## Competing interests

The authors declare no competing interest.

## Authors’ contributions

LSM, AC and MB conceptualized and designed the study, LSM collected the data, LSM, MT, RN and EOO analyzed the data and drafted the manuscript. All authors read and approved the final manuscript.

## Pre-publication history

The pre-publication history for this paper can be accessed here:

http://www.biomedcentral.com/1472-6831/13/12/prepub
